# Treatment of Erysipelas

**Published:** 1873-01

**Authors:** 


### TREATMENT OF ERYSIPELAS.

In the monthly supplement to the Allgemeine Wiener Medizinische Zeitung for July, there is notice of Wilde's treatment of erysipelas. He holds that this malady is produced by the presence of some low vegetable growth, *e. g.*, "micrococcus" or "monads," and that this parasite superinduces a "*capillar-lymphangiotis*" of the skin. It is natural, on this view, that the treatment should resolve itself into an effort to destroy the enemy *in situ.* This, according to Wilde, must be done by the subcutaneous injection of some solution destructive to fungoid life. He rejects carbolic acid for this purpose, on account of its irritating properties, and substitutes for it the lately recommended sulpho-carbolate of soda. This is dissolved in water in the proportion of one to twelve, and injected under the skin. There is little or no pain, and the effect, according to Wilde, is constant and most striking. On the third or fourth day at latest, there is nothing of the erysipelas recognizable, except that the skin is a little œdematous. Wilde has also favorably employed this solution in diphtheria.

In the Abeille Médicale for the 12th of August, Professor Broca recommends the application of collodion in erysipelas. He would lay it all around the part attacked, extending it somewhat over the healthy skin. Any fissures which may occur in it ought to be immediately repaired. The slight circular compression which is thus exercised seems to arrest the march of the disease. There must be no oil in the collodion.—*Doctor.*

